# Genetic Environment of Plasmid Mediated CTX-M-15 Extended Spectrum Beta-Lactamases from Clinical and Food Borne Bacteria in North-Eastern India

**DOI:** 10.1371/journal.pone.0138056

**Published:** 2015-09-11

**Authors:** Supriya Upadhyay, Abbas Hussain, Shweta Mishra, Anand Prakash Maurya, Amitabha Bhattacharjee, Santa Ram Joshi

**Affiliations:** 1 Department of Biotechnology & Bioinformatics, North Eastern Hill University, Shillong, Meghalaya, India; 2 Department of Microbiology, Institute of Medical Sciences, Banaras Hindu University, Varanasi, India; 3 Department of Microbiology, Assam University, Silchar Assam, India; Amphia Ziekenhuis, NETHERLANDS

## Abstract

**Background:**

The study investigated the presence of CTX-M-15 type extended spectrum beta-lactamases (ESBL), compared their genetic arrangements and plasmid types in gram negative isolates of hospital and food origin in north-east India. From September 2013 to April 2014, a total of 252 consecutive, non-duplicate clinical isolates and 88 gram negative food isolates were selected. Phenotypic and molecular characterization of ESBL genes was performed. Presence of integrons and gene cassettes were analyzed by integrase and 59 base-element PCR respectively. The molecular environments surrounding *bla*_CTX-M_ and plasmid types were investigated by PCR and PCR-based replicon typing respectively. Transformation was carried out to assess plasmid transfer. Southern blotting was conducted to localize the *bla*_CTX-M-15_ genes. DNA fingerprinting was performed by ERIC-PCR.

**Results:**

Prevalence of ESBL was found to be 40.8% (103/252) in clinical and 31.8% (28/88) in food-borne isolates. Molecular characterization revealed the presence of 56.3% (58/103) and 53.5% (15/28) *bla*_CTX-M-15_ in clinical and food isolates respectively. Strains of clinical and food origin were non-clonal. Replicon typing revealed that IncI1 and IncFII plasmid were carrying *bla*_CTX-M-15_ in clinical and food isolates and were horizontally transferable. The IS*Ecp*1 element was associated with *bla*_CTX-M-15_ in both clinical and food isolates.

**Conclusions:**

The simultaneous presence of resistance determinants in non-clonal isolates of two different groups thus suggests that the microbiota of common food products consumed may serve as a reservoir for some of the drug resistance genes prevalent in human pathogens.

## Introduction

Extended spectrum beta-lactamases conferring resistance to third generation cephalosporins in Gram negative bacteria is a global health threat. In hospital settings infection with ESBL-producing organisms results in poor clinical outcomes, delay in antibacterial therapy, longer hospital stay and increased hospital expenses [1]. Thus, these organisms serve as a potential threat and economic burden for the public health departments and communities. Over the past decade CTX-M has become the most prevalent family of ESBLs encoded by *bla*_CTX-M._ More than 147 different variants of CTX-M have been reported (http://www.lahey.org/studies), of which CTX-M-15 is the most predominant one, first reported from the Indian subcontinent in 2001 and expanded worldwide [2, 3]. Since then numerous reports documented the presence of *bla*_CTX-M-15_ among nosocomial isolates in this subcontinent [4]. The incidence of infections due to such resistant organisms has rapidly increased over the last decade and has become a worldwide epidemic. Besides their resistance to cephalosporins these organisms often display co-resistance to aminoglycosides, tetracyclines and sulfonamides posing serious therapeutic challenge [5].

To make things worse, *bla*_CTX-M-15_ is found on plasmid and other mobile genetic elements [2] and is horizontally transferred to other pathogenic bacterial strains or can even cross species barrier. Various types of genetic environments might be involved in the mobilization of *bla*_CTX-M_ genes, of which IS*Ecp*1 (Insertion sequence) is often associated upstream of the *bla*_CTX-M_ genes, hence serving as an efficient tool for the lateral transfer and expression [2, 6– 8]. In addition, several different insertion sequences *viz*. IS*26*, IS*10*, IS5 and IS*903* have been detected surrounding the resistance determinant [6, 9–11].

For isolates harbouring *bla*_CTX-M-15_, their genetic linkage and transmission dynamics have been reported frequently and very well documented in the nosocomial isolates whereas; in the community numerous reservoirs have been reported which involve non-human pathogens like poultry, food-producing animals, pets and also the raw vegetables [12–15]. But there is paucity of data pertaining to the persistence and genetic arrangement of *CTX-M* gene among food-borne isolates. Therefore, it becomes necessary to evaluate if the ready to eat foods available at the stalls and eating outlets or raw food may constitute as reservoir and contribute to the rapid expansion of ESBLs. Also, it is important to know the factors that are involved in maintenance of these determinants in the food isolates and potential risk of transmission of these resistant organisms through the food chain in the environmental conditions.

Thus, present study was undertaken to document the prevalence of *bla*_CTX-M-15_ type ESBL genes, their genetic arrangement and transmissibility among nosocomial isolates as well as in food-borne isolates obtained from food samples available at eating outlets in north-east India.

## Materials and Methods

### Bacterial Strains

A total of 252 consecutive, non-duplicate, gram negative rods [*Escherichia coli* (n = 178), *Klebsiella pneumoniae* (n = 25), *Klebsiella oxytoca* (n = 7), *Citrobacter freundii* (n = 11), *Proteus mirabilis* (n = 5), *Proteus vulgaris* (n = 2), *Salmonella typhi* (n = 1), *Enterobacter aerogenes* (n = 4), *Enterobacter cloacae* (n = 2), *Morganella morganii* (n = 1), *Pseudomonas aeruginosa* (n = 16)] were collected from different clinical specimens during September 2013 to April 2014 from different wards/clinics of various hospitals (Nazareth Hospital, The Children’s Hospital, and Pasteurs Institute) of Shillong, India. Subjects included in the present study were diagnosed as a case of one of the following; meningitis, bacterial pneumonia, urinary tract infection, pyogenic infection, bacteremia/septicemia or diarrheal diseases (S1 File. Method of bacterial strain collection). Clinical samples obtained were inoculated onto the MacConkey agar plates and incubated. All lactose fermenting and nonfermenting colonies with different coloration and morphology were picked from the selective plates, subcultured and stored in Glycerol stock (15%) at -80°C.

Apart from clinical strains, a total of 88 gram negative bacteria [*E*. *coli* (n = 48), *K*. *pneumoniae* (n = 14), *Klebsiella* spp. (n = 3), *Citrobacter* spp. (n = 3), *P*. *mirabilis* (n = 7), *E*. *aerogenes* (n = 2), *E*. *cloacae* (n = 5) *Pseudomonas* spp. (n = 6)] were isolated from variety of cooked (cakes, sweets, ice-creams etc) and uncooked (vegetables, meat) food samples (Table 1). Samples were collected using sterile vials from various local sweet shops, meat shops, street vendors and other eating outlets situated in remote as well as at the prime locations of the Shillong city from September 2013 to April 2014. Sterile cotton gauze was used to swab properly all over the surface of samples. For further analysis, serial dilutions were prepared starting with 1 mg of food samples diluted in 9ml of saline solution (0.9% NaCl). A volume of 100μl from each well homogenized dilution was inoculated onto the MacConkey agar containing vancomycin (3mg/L). Plates were incubated at 37°C for 24 h under aerobic conditions. All lactose fermenting and non-lactose fermenting colonies with different coloration and morphology belonging to the members of enterobacteriaceae family as well as non fermenting gram negative rods were picked from the selective plates, subcultured and stored. All the isolates were identified according to Gram stain, cultural characteristics and biochemical reactions [16].

**Table 1:** Distribution of ESBL among different samples.

Isolates	Total number of GNB (N)	ESBL n/N (%)	*bla*_CTX-M-15_	Upstream IS*Ecp*
Clinical samples	252	103/252 (40.8%)	58/103 (56.3%)	37
Food samples	88	28/88 (31.8%)	15/28 (53.5%)	8
Total	340	131/340 (38.5%)	73/131 (59.5%)	45

GNB = Gram Negative Bacilli; N = Total number of GNB; n = total number of ESBL positive strains

### Ethical approval

The work was approved by Institutional Ethics Committee for Human Samples/Participants of North Eastern Hill University, vide reference number: IECHSP/2014/03. The authors confirm that all the patients, patients’ parents/guardians provided their written informed consent to participate in the study. Written permission was obtained from the owners of the shops for carrying out experiment from their food samples. The authors hereby also confirm that the field study did not involve any protected or endangered species.

### Phenotypic detection of ESBL

All the isolates were selected on the basis of their initial screening followed by the confirmatory combined disc diffusion method for presence of ESBL according to CLSI recommendation [17].

### Molecular characterization of *bla*_ESBL_ genes by Multiplex PCR

DNA was extracted by the boiling centrifugation method. To determine the genotypes of ESBLs, PCR was performed using primers specific to *bla*_TEM_, *bla*_CTX-M_, *bla*_SHV_, *bla*_OXA-2_, *bla*_OXA-10_ and *bla*_GES_ as described previously [18]. Reaction mixture was prepared using Promega 2X PCR mix (Promega, Madison, USA). Reactions were run under the following conditions: initial denaturation 94°C for 5 min, 32 cycles of 94°C for 1 min, 54°C for 1min, 72°C for 1 min and final extension at 72°C for 7 min. PCR amplification was performed with a PCR System 9700 (Applied Biosystems, USA). The amplicons were sequenced and compared by performing BLAST (http://blast.ncbi.nlm.nih.gov/Blast.cgi). Three previously confirmed isolates of *E*. *coli* producing *bla*_TEM,_ *bla*_CTX-M_, *bla*_SHV_ were taken as positive control while *E*. *coli* ATCC 25922 was used as negative control in the PCR reaction.

### Multiplex PCR assay for AmpC beta lactamases and carbapenemases encoding genes

All the ESBL positive isolates were tested for presence of plasmidic AmpC [19] and class A and B carbapenemases [20–23]. Reactions conditions were as described previously [19–23]

### Genetic environment of *bla*_CTX-M-15_

Integrase gene PCR was performed for the characterization of class I and class II integrons, using IntI F’ CAGTGGACATAAGCCTGTTC, IntI R’ CCCGAGGCATAGACTGTA and IntII F’ TTGCGAGTATCCATAACCTG, IntII R’ TTACCTGCACTGGATTAAGC primers [24]. For association of *bla*_CTX-M_ with other gene cassettes, 59 base element (59be) PCR was performed using HS 286 and HS 287 primers [24]. In order to determine the variable regions of integron gene cassettes carrying *bla*_CTX-M,_ cassette PCR was performed amplifying 5’ and 3’ conserved sequences [25]. Two reactions were performed consequently: in one reaction 5CS and reverse primer of *bla*_CTX-M_ and in other reaction 3CS and forward primer of *bla*_CTX-M_ was used [18, 25]. PCR reactions were run under the following conditions: initial denaturation 95°C for 2 min, 29 cycles of 95°C for 20 sec, 54°C for 1 min, 72°C for 2 min and final extension at 72°C for 10 min.

### Genetic association of the CTX-M gene with insertion sequence IS*Ecp*1

To determine the association of *bla*_CTX-M_ with IS*Ecp*1, PCR analysis was performed using the forward primer for IS*Ecp*1 (5'-AAAAATGATTGAAAGGTGGT-3') and the reverse primer for CTX-M gene (5’-ACCGCGATATCGTTGGT-3’). The reaction mixture and running conditions were same as described previously for gene cassettes.

### Sequencing Analysis

All the amplicons were sequenced (Xcelris Labs, India) and compared by performing BLAST (http://blast.ncbi.nlm.nih.gov/Blast.cgi).

### Plasmid preparation

All *bla*_CTX-M_ producing bacterial isolates were cultured in Luria Bertani broth (Hi-Media, Mumbai, India) containing 1mg/L of cefotaxime. Plasmids were purified by standard alkaline lysis method.

### Southern hybridization for determination of *bla*_CTX-M-15_ carriage

To validate our study, Southern blotting was performed on agarose gel by in-gel hybridization [26] with the *bla*_CTX-M-15_ probe labelled with Dig High Prime Labeling Mix (Roche, Germany) detection Kit. The dig-oxigenin-labeled *bla*_CTX-M-15_ specific probe was prepared using primers (CTX-M-15 Forward 5’ CGCTTTGCGATGTGCAG3’ and Reverse 5’ ACCGCGATATCGTTGGT 3’) that amplify a 550 bp region of the *bla*_CTX-M_. Separated plasmid DNA on agarose gel was transferred to nylon membrane (Hybond N, Amersham, UK) and then hybridised with prepared *bla*_CTX-M_ specific probe. Detection was performed by using an NBT color detection kit (Roche, Germany).

### Gene Transferability of *bla*_ESBL_ gene by transformation

Transformation was carried out using *E*. *coli* DH5α as recipient. Transformants were selected on cefotaxime (0.5 mg/L) containing LB Agar plates.

### Plasmid stability and incompatibiltiy typing

Plasmid stability of *bla*_CTX-M_ producers as well as their transformants were analyzed by serial passages method for consecutive 75 days at 1:1000 dilutions in LB broth without antibiotic pressure as described previously [27]. PCR for presence of the *bla*_CTX-M-15_ was carried out for the isolates after each passage. Incompatibility typing was carried out by PCR based replicon typing [28] among all the transformants carrying *bla*_CTX-M._

### Antimicrobial susceptibility and minimum inhibitory concentrations (MIC) determination

Antimicrobial susceptibility of *bla*_CTX-M_ harbouring parent strains as well as transformants were determined by Kirby Bauer disc diffusion method and results were interpreted as per CLSI guidelines [17]. Following antibiotics were tested: cefotaxime (30μg), cefoxitin (30μg), ceftazidime (30μg), amikacin (30μg), gentamicin (10μg), kanamicin (30μg), ciprofloxacin (5μg), trimithoprim/sulphamethoxazole (1.25/23.75μg), imipenem (10μg), ertapenem (10μg), tigecycline (15μg) and polymyxin B (300 units) (Hi-Media, Mumbai). MIC was also determined for donor strain and transformants against cefotaxime, ceftazidime and ceftriaxone (Hi-Media, Mumbai, India) by agar dilution method.

### Typing of isolates

Enterobacterial repetitive intergenic consensus (ERIC) PCR was performed for typing of all the *CTX-M* harbouring isolates [29].

## Results

Among the tested strains, ESBL production was obtained in 103 (40.8%) clinical and 28 (31.8%) food isolates (S1 Table). Molecular characterization revealed presence of 56.3% (58/103) *bla*_CTX-M_, 12.6% (13/103) *bla*_TEM_ and 9.7% (10/103) *bla*_SHV_ in clinical isolates (S2 Table), while 53.5% (15/28) food isolates showed the presence of CTX-M gene (Table 2). Few clinical isolates also revealed presence of multiple ESBL genes, while some ESBL producers (n = 27) did not show any amplifications by the primers used in the study. Among studied genes, *bla*_CTX-M_ was found to be predominantly present in both clinical as well as food isolates. Distribution of CTX-M gene in different clinical isolates was as follows: *E*. *coli* (n = 45), *K*. *pneumoniae* (n = 6), *P*. *mirabilis* (n = 1), *P*. *aeruginosa* (n = 6). Among food borne isolates, *CTX-M* was present in *E*. *coli* (n = 8), *K*. *pneumoniae* (n = 4), *Citrobacter* spp. (n = 3). Sequencing of *bla*_CTX-M_ PCR products revealed the presence of *bla*_CTX-M-15_ variant among all the isolates whereas SHV-22 and TEM-1 (non-ESBL) too were confirmed by sequencing. Carriage of *ampC* was also observed in significant number of isolates (33 in clinical and 4 in food isolates). *bla*_CIT_ type was predominant one (n = 32) followed by *bla*_EBC_ (n = 5). No carbapenemase gene could be detected by the PCR. On observing DNA fingerprinting results in case of clinical isolates, 28 patterns of *E*. *coli*, 4 patterns of *K*. *pneumoniae* and 5 patterns of *P*. *aeruginosa* were obtained. Among food isolates 4 patterns of *E*. *coli* and 3 patterns of *K*. *pneumoniae* were observed. There was no clonal similarity between clinical and food borne isolates by ERIC-PCR.

**Table 2:** Molecular characterization of *bla*_CTX-M-15_ harbouring food borne isolates.

Food samples	Total GNB	ESBLs	CTX-M-15	PBRT
Raw Chicken	22	8	5	IncFII (4) Untypeable (1)
Raw mutton	16	5	4	IncFII (4)
Raw Fish	15	3	1	Untypeable (1)
Salad	9	2	1	IncFII (1)
Pancake	5	3	2	IncI1 (2)
Pastry/cake	8	3	0	0
Sweets	7	4	2	IncI1 (2)
Jaljeera water	2	0	0	0
Icecream	4	0	0	0
Total	88	28	15	15

GNB: Gram negative bacilli; PBRT: PCR based replicon typing

On observing the antimicrobial susceptibility of the *CTX-M* encoding isolates, 100% susceptibility was shown towards tigecycline and polymyxin B followed by imipenem (95.8%), ertapenem (95.1%) and amikacin (89.6%), while all other antibiotics including third generation cephalosporins, aminoglycosides and flouroquinolones showed moderate to poor activity (Table 3). In comparison to food isolates, most of the clinical isolates showed multidrug-resistant phenotype. A high MIC in the resistant range was observed against all tested cephalosporins (Table 4).

**Table 3:** Antibiotic susceptibilities for CTX-M positive isolates obtained from hospital and food samples.

Antimicrobial agent	Clinical isolates n = 58 (%)	Food isolates n = 15 (%)
Cefoxitin	13.7	40.0
Cefotaxime	3.4	26.6
Ceftazidime	5.1	40.0
Imipenem	95.8	100
Ertapenem	95.1	100
Amikacin	89.6	93.3
Gentamicin	75.8	80.0
Kanamycin	50.0	91.6
Co-trimoxazole	15.5	26.6
Ciprofloxacin	36.2	60.0
Tigecycline	100	100
Polymixin B	100	100

**Table 4:** MIC_50_ and MIC_90_ (mg/L) of *bla*_CTX-M-15_ harboring clinical and food-borne isolates and their transformants.

Antibiotics	Cefotaxime		Ceftazidime		Ceftriaxone	
	MIC_50_	MIC_90_	MIC_50_	MIC_90_	MIC_50_	MIC_90_
Clinical isolates (n = 58)	256	≥256	256	≥256	256	≥256
Food borne isolates (n = 15)	128	≥256	64	256	128	≥256
Transformants (n = 35)	128	128	64	128	128	128

n = number of strains tested for MIC

Transferability assay showed that *CTX-M* could be horizontally transferred from diverse host range to recipient *E*. *coli* DH5α. However, plasmids of *P*. *aeruginosa* could not replicate within *E*. *coli* host. The plasmids of the transformants could be hybridized with *CTX-M-15* specific probe. In replicon typing, it was observed that *CTX-M-15* was carried within Inc I1 and Inc F II types. In nine transformants, the plasmid was untypable. All the *CTX-M* positive isolates were found to harbour a class I integron. Plasmids in clinical isolates were more stable than those of food isolates where plasmid carrying *CTX-M* was retained after 75 passages for clinical isolates while in case of food isolates plasmid was lost after 62 serial passage. However, transformants did not show high stability as plasmids were lost between 15 to 36 passages.

**Fig 1:**
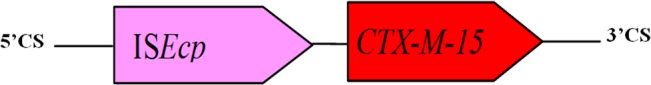
Schematic diagram showing the genetic location of IS*Ecp* upstream of *bla*_CTX-M-15._ The arrows indicate the direction of transcription.

**Fig 2:**
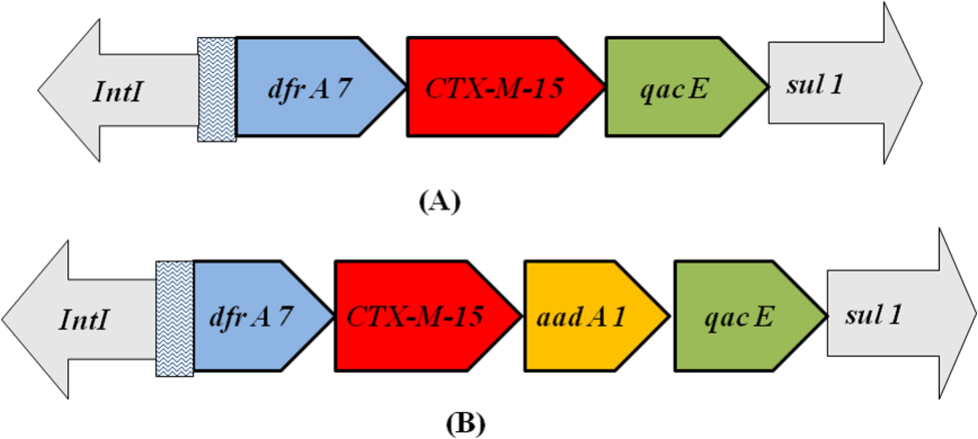
Schematic diagram showing the genetic environment of *bla*_CTX-M-15_ on gene cassette with other resistance determinants (A, B). The arrows indicate the direction of transcription.

*CTX-M-15* was associated with IS*Ecp*1 in the upstream region in 37 clinical and 8 food isolates (Fig 1). Among the rest of isolates, interestingly, integron mediated *CTX-M-15* was observed particularly in food isolates. Two different arrangements were observed along with other resistance determinants (Fig 2).

## Discussion

The present study demonstrated both the hospital and community based transmission of *bla*_CTX-M-15_, raising a threat to public health. The study simultaneously analyzed the CTX-M-15 type ESBL genes, their plasmid type and genetic environment from food borne and human clinical Gram negative isolates from this part of the world. CTX-M-15 is one of the most widespread ESBL genes and has significant impact on the treatment of hospital and community acquired infections. Majority of the *bla*_CTX-M-15_ harbouring nosocomial isolates investigated in the present study were obtained from the females diagnosed with urinary tract infection (UTI). Apart from female patients, the gene was also reported in immuno-suppressed patients of intensive care unit as well as from the out-patient departments. It is well established that such resistant organisms can be frequently transmitted to a wide range of geographical region. Similar to the present findings, several other studies reported the high rate of ESBL producers among UTI patients [30, 31]. The study further demonstrated the presence of *CTX-M* encoding isolates in raw meat and in ready-to-eat foods like salad, cakes and sweets which significantly increases their dissemination and colonization in gut flora. The incidence of *CTX-M-15* harbouring isolates in food could serve as potential reservoir of pathogens prevalent in hospital settings. Such situations pose high risk of exposure of healthy humans to these multidrug resistant strains during handling and consumption of meat, sweets and vegetables and may contribute to the colonization of these strains in human gut along with commensals. This suggests that, there should be increased awareness amongst such food handlers regarding the safe and hygienic practices. In contrast to our findings, previous reports from Aligarh, India and from Northern Ireland depicted the dissemination of *bla*_CTX-M_ in clinical isolates but none of the food borne isolates was harbouring the gene [32, 33]. While, similar to our report, a study from Spain in 2003 described *CTX-M* encoding gram negative isolates in cooked foods, salads and raw chicken [34]. In this study, around 37% of *CTX-M-15* harbouring isolates with cefotaxime susceptibility might be attributed to heterogenic resistance property. In the past, reports have also shown similar oxy-imino cephalosporin susceptibility among ESBL producers [35]. Presence of AmpC beta lactamase in our study isolates might be responsible for exhibiting carbapenem nonsusceptibility in few isolates possibly with porin loss as no carbapenamase gene could be traced by PCR assay.

This study advocates diverse source of origin and acquisition of *CTX-M-15* in the study area, as the genetic vehicle for transfer of this gene were the two different Inc type plasmids. Similar to earlier studies, *bla*_CTX-M-15_ was located on replicon type Inc FII whereas other replicon type Inc I1 was also reported [31, 36]. Further, IS*Ecp*1 may be an additional efficient tool for the enhanced mobilization and expression of *bla*_CTX-M-15_ which corroborates to previous reports [2, 6–8]. In the present study, presence of IS*Ecp*1 in the upstream region of *bla*_CTX-M-15_ across the species indicates that the whole insertion sequence or a part of it possibly excised along with *CTX-M-15* during horizontal transfer. Integrons play an important role in the dissemination of these resistance determinants among bacterial flora. Another aspect of this study was the location of *bla*_CTX-M-15_ on gene cassettes along with other resistance genes which includes *dfr7*, *aadA1*, *sul1*, which enhances the risk of co-selection. The genetic arrangement of *CTX-M-15* with IS*Ecp*1 and integrons highlights the role of these mobile elements in the maintenance of this gene in the environment where the antibiotic pressure is invariably considered less compared to hospital settings. The study could highlight a new genetic background of CTX-M—ESBL. This particular finding may be well explained as a reason for expansion of these resistance determinants both vertically and horizontally with better stability and their spread does not confine to a particular geographical location. This study also puts forward the concern for potential threat for treatment failure and maintenance of cephalosporin resistance in a given global situation.

## Conclusions

Indiscriminate use, over the counter availability and unethical marketing of antibiotics has already created havoc for treating common infections. Additionally, contamination of foods by food handlers particularly ESBL carriers warrant urgent hygiene measures to slow down the spread of resistance. This demands a need to increase the awareness amongst these food handlers regarding the safe and hygienic practices. In a country with already existing burden of antibiotic pressure in hospital settings, presence and propagation of these resistance determinants in the food borne isolates could serve as an additional reservoir. Transmission of these determinants to healthy individuals, augment the grave situation and contributes to the emergence of drug resistant pathogens in this location. Considering this fact, the presence and persistence of resistance determinants (CTX-M-15) in food isolates underscores the potential threat of their spread within pathogens thereby restricting therapeutic alternatives.

## Supporting Information

S1 File Method of bacterial strain collection. (DOCX)

S1 Table Details of isolates obtained from food samples. (DOCX)

S2 Table Clinical details of ESBL gene harbouring isolates. (DOCX)
