# Supplementary material for: Genetic Environment of Plasmid Mediated CTX-M-15 Extended Spectrum Beta-Lactamases from Clinical and Food Borne Bacteria in North-Eastern India
Source: PLoS One. 2015 Sep 11;10(9):e0138056. doi: 10.1371/journal.pone.0138056 (PMC4567302; doi:10.1371/journal.pone.0138056)
Supplement: S1 File — (DOCX) [file pone.0138056.s001.docx]

**S1 File. Method of bacterial strain collection.**

The patients who were admitted to different wards and those who attended the outpatient departments of Nazareth Hospital, Pasteur Institute and The Children Hospital, were selected for the study. Subject included in the present study were diagnosed as a case of one of the following; meningitis, bacterial pneumonia, urinary tract infection, pyogenic infection, bacteremia/septicemia or diarrheal diseases.

The clinical specimens were collected from those patients only who visited the hospital for their treatment and further the microbiological analysis was suggested by physicians of hospital, also the written consent was obtained from the patients parents/guardians for their participation in the study plan. All the clinical samples were collected and processed by the authorized and well trained personnel of the hospitals. The gram negative bacilli isolated from such clinical specimens were obtained from the hospital for the study. A duplicate copy of bacterial strain obtained from hospitals and were inoculated onto the MacConkey agar plates and incubated. All lactose fermenting and nonfermenting colonies with different coloration and morphology were picked from the selective plates, subcultured and stored in Glycerol stock (15%) at -80 °C.
