# Supplementary material for: Genetic Environment of Plasmid Mediated CTX-M-15 Extended Spectrum Beta-Lactamases from Clinical and Food Borne Bacteria in North-Eastern India
Source: PLoS One. 2015 Sep 11;10(9):e0138056. doi: 10.1371/journal.pone.0138056 (PMC4567302; doi:10.1371/journal.pone.0138056)
Supplement: S1 Table — (DOCX) [file pone.0138056.s002.docx]

**S1 Table. Details of isolates obtained from food samples.**

| **S.No.** | **Strain ID** | **Organism isolated** | **Food sample** | **Date of isolation** | **Place of isolation** | **Shop name** | **ESBL, if any** |
| --- | --- | --- | --- | --- | --- | --- | --- |
| 1 | FS-Ec 1 | *E. coli* | Raw chicken | September 2013 | Polo, Shillong | Retail Meat Shop | CTX-M |
| 2 | FS-Ec 3 | *E. coli* | Raw chicken | Sept 2013 | Polo, Shillong | Retail meat shop | CTX-M |
| 3 | FS-Ec 7 | *E. coli* | Raw chicken | Jan 2014 | Police bazaar, Shillong | Local meat shop | Phenotypically positive for ESBL |
| 4 | FS-Ec 10 | *E. coli* | Raw chicken | Sept 2013 | Polo, Shillong | Meat shop | CTX-M |
| 5 | FS-Ec 19 | *E. coli* | Raw mutton | Jan 2014 | Garikhana, Shillong | Meat store | CTX-M |
| 6 | FS-Ec 22 | *E. coli* | Raw mutton | Jan 2014 | Polo, Shillong | Roadside meat stall | CTX-M |
| 7 | FS-Ec 27 | *E. coli* | Raw Fish | Jan 2014 | Zail road, Shillong | Fish stall | Phenotypically positive |
| 8 | FS-Ec 31 | *E. coli* | Pastry/cake | Feb 2014 | Police bazaar, Shillong | Local meat store | Phenotypically positive |
| 9 | FS-Ec 35 | *E. coli* | Salad | Feb 2014 | Police bazaar, Shillong | Street stall | CTX-M |
| 10 | FS-Ec 36 | *E. coli* | Pancake | Dec 2013 | Mawlai, Shillong | Mobile food van | Phenotypically positive |
| 11 | FS-Ec 40 | *E. coli* | Pancake | Dec 2014 | Police bazaar, Shillong | Mobile food van | CTX-M |
| 12 | FS-Ec 42 | *E. coli* | Sweets | April 2014 | Rynjah, Shillong | Sweet shop | Phenotypically positive |
| 13 | FS-Ec 46 | *E. coli* | Sweets | April 2014 | Polo, Shillong | Sweet shop | CTX-M |
| 14 | FS-Kp 3 | *K. pneumoniae* | Raw chicken | Feb 2014 | Zail Road, Shillong | Meat shop | CTX-M |
| 15 | FS-Kp 4 | *K. pneumoniae* | Raw chicken | Feb 2014 | Zail road, Shillong | Retail meat store | Phenotypically positive |
| 16 | FS-Kp 7 | *K. pneumoniae* | Raw mutton | Feb 2014 | Polo, Shillong | Chicken and mutton retail shop | CTX-M |
| 17 | FS-Kp 9 | *K. pneumoniae* | Raw mutton | March 2014 | Garikhana, Shillong | Meat retail shop | Phenotypically positive |
| 18 | FS-Kp10 | *K. pneumoniae* | Raw fish | March 2014 | Laitumkrah, Shillong | Fish retail shop | Phenotypically positive |
| 19 | FS-Kp11 | *K. pneumoniae* | Pancake | Jan 2014 | Polo, Shillong | Bus stop canteen | CTX-M |
| 20 | FS-Kp13 | *K. pneumoniae* | Sweets | Oct 2013 | Polo, Shillong | Sweet shop | CTX-M |
| 21 | FS-Cs 1 | *Citrobacter* spp. | Raw chicken | Sept 2013 | Polo, Shillong | Retail meat shop | CTX-M |
| 22 | FS-Cs 2 | *Citrobacter* spp. | Raw mutton | Feb 2014 | Polo, Shillong | Chicken and mutton retail shop | CTX-M |
| 23 | FS-Cs 3 | *Citrobacter* spp. | Raw fish | Feb 2014 | Rynjah, Shillong | Street stall | CTX-M |
| 24 | FS-Ecl 3 | *E. cloacae* | Sweets | March 2014 | Mawlai, Shillong | Streetside canteen | Phenotypically positive |
| 25 | FS-Ecl 5 | *E. cloacae* | Salad | Feb 2014 | PB Shillong | Street stall | Phenotypically positive |
| 26 | FS-Pm 1 | *P. mirabilis* | Raw chicken | April 2014 | Mawlai, Shillong | Meat store | Phenotypically positive |
| 27 | FS-Pm 4 | *P. mirabilis* | Raw mutton | April 2014 | Mawlai, Shillong | Meat store | Phenotypically positive |
| 28 | FS-Pm 7 | *P. mirabilis* | Pastry/cake | April 2014 | Laban, Shillong | Local pastry shop | Phenotypically positive |
