# Supplementary material for: Genetic Environment of Plasmid Mediated CTX-M-15 Extended Spectrum Beta-Lactamases from Clinical and Food Borne Bacteria in North-Eastern India
Source: PLoS One. 2015 Sep 11;10(9):e0138056. doi: 10.1371/journal.pone.0138056 (PMC4567302; doi:10.1371/journal.pone.0138056)
Supplement: S2 Table — (DOCX) [file pone.0138056.s003.docx]

**S2 Table. Clinical details of ESBL gene harbouring isolates.**

| **SN.** | **Strain ID** | **Place of isolation** | **Organism isolated** | **Ward/OPD** | **Clinical specimen** | **Patient age/sex** | **ESBL gene** |
| --- | --- | --- | --- | --- | --- | --- | --- |
| 1 | NH-Ec 2 | Nazareth Hospital | *E.coli* | Urology | Urine | 47 years/Female | TEM |
| 2 | NH-Ec 4 | NH | *E.coli* | Urology | Urine | 33yr/Female | CTX-M |
| 3 | NH-Ec 5 | NH | *E.coli* | Urology | Urine | 21yr/ Female | CTX-M |
| 4 | NH-Ec 6 | NH | *E.coli* | OPD | Urine | 55yr/ Female | CTX-M |
| 5 | NH-Ec 8 | NH | *E.coli* | OPD | Urine | 20 yr/Female | CTX-M |
| 6 | NH-Ec 11 | NH | *E.coli* | CHW | Stool | 7 yr/Female | CTX-M |
| 7 | NH-Ec 12 | NH | *E.coli* | FMW | Stool | 71yr/Female | CTX-M |
| 8 | NH-Ec 13 | NH | *E.coli* | FMW | Urine | 37yr/Female | CTX-M |
| 9 | NH-Ec 14 | NH | *E.coli* | Urology | Urine | 20yr/Female | CTX-M |
| 10 | NH-Ec 16 | NH | *E.coli* | NICU | Urine | 8days/Male | CTX-M |
| 11 | NH-Ec 18 | NH | *E.coli* | OPD | Urine | 25yr/Female | CTX-M |
| 12 | NH-Ec 25 | NH | *E.coli* | CHW | Urine | 1yr/Male | CTX-M |
| 13 | NH-Ec 26 | NH | *E.coli* | FMW | Urine | 23yr/Female | CTX-M |
| 14 | NH-Ec 29 | NH | *E.coli* | OPD | Urine | 16Yr/Female | CTX-M |
| 15 | NH-Ec 31 | NH | *E.coli* | ICU | CSF | 52yr/Male | CTX-M, TEM |
| 16 | NH-Ec 39 | NH | *E.coli* | OPD | Urine | 48yr/Female | CTX-M |
| 17 | NH-Ec 40 | NH | *E.coli* | FSW | Urine | 19yr/Female | CTX-M |
| 18 | NH-Ec 41 | NH | *E.coli* | MSW | CSF | 48yr/Male | TEM |
| 19 | NH-Ec 45 | NH | *E.coli* | OPD | Urine | 29yr/Female | TEM |
| 20 | NH-Ec 47 | NH | *E.coli* | Urology | Urine | 30yr/Female | CTX-M |
| 21 | NH-Ec 49 | NH | *E.coli* | Urology | Stool | 42yr/Male | CTX-M |
| 22 | NH-Ec 51 | NH | *E.coli* | CHW | Blood | 1yr/Male | CTX-M |
| 23 | NH-Ec 56 | NH | *E.coli* | OPD | Urine | 34yr/Female | CTX-M |
| 24 | NH-Ec 58 | NH | *E.coli* | FSW | Urine | 14yr/Female | CTX-M |
| 25 | NH-Ec 62 | NH | *E.coli* | CHW | Stool | 6yr/Male | CTX-M |
| 26 | NH-Ec 64 | NH | *E.coli* | Urology | Urine | 24yr/Female | CTX-M |
| 27 | NH-Ec 69 | NH | *E.coli* | FSW | Urine | 26yr/Female | CTX-M |
| 28 | NH-Ec 70 | NH | *E.coli* | MW | Urine | 19yr/Female | CTX-M |
| 29 | NH-Ec 71 | NH | *E.coli* | OPD | Urine | 32yr/Female | CTX-M |
| 30 | NH-Ec 72 | NH | *E.coli* | FMW | Pus | 72yr/Female | CTX-M |
| 31 | NH-Ec 74 | NH | *E.coli* | CHW | Blood | 3yr/Female | CTX-M |
| 32 | NH-Ec 75 | NH | *E.coli* | CHW | Pus | 1yr/Male | CTX-M |
| 33 | NH-Ec 76 | NH | *E.coli* | ICU | Tracheal aspirate | 10yr/Male | CTX-M |
| 34 | NH-Ec 78 | NH | *E.coli* | OPD | Urine | 57yr/Female | CTX-M |
| 35 | NH-Ec 84 | NH | *E.coli* | MW | Blood | 25yr/Female | CTX-M |
| 36 | NH-Ec 86 | NH | *E.coli* | MW | Pus | 50yr/Female | CTX-M |
| 37 | NH-Ec 93 | NH | *E.coli* | FMW | Blood | 67yr/Female | CTX-M |
| 38 | CH-Ec 5 | Children Hospital | *E.coli* | NICU | Tracheal aspirate | 1 week/Male | CTX-M |
| 39 | CH-Ec 9 | CH | *E.coli* | OPD | Sputum | 5 yr/Female | CTX-M |
| 40 | CH-Ec 17 | CH | *E.coli* | CHW | Blood | 8yr/Male | CTX-M |
| 41 | CH-Ec 23 | CH | *E.coli* | CHW | Urine | 2yr/Female | CTX-M |
| 42 | CH-Ec 25 | CH | *E.coli* | SW | Stool | 2yr/Female | CTX-M |
| 43 | CH-Ec-31 | CH | *E.coli* | NICU | Urine | 10daysr/Male | CTX-M |
| 44 | CH-Ec 32 | CH | *E.coli* | Urology | Sputum | 8yr/Male | CTX-M |
| 45 | CH-Ec 35 | CH | *E.coli* | ICU | Blood | 11yr/Male | CTX-M |
| 46 | PI-Ec 6 | Pasteur Institute | *E.coli* | FSW | Pus | 29yr/Female | CTX-M |
| 47 | PI-Ec 10 | PI | *E.coli* | SW | Pus | 38yr/Male | CTX-M |
| 48 | PI-Ec 11 | PI | *E.coli* | ICU | Tracheal Aspirate | 46yr/Male | CTX-M |
| 49 | PI-Ec 13 | PI | *E.coli* | OPD | Urine | 1yr/Male | TEM |
| 50 | PI-Ec 14 | PI | *E.coli* | FSW | Pus | 5yr/Female | TEM |
| 51 | PI-Ec 17 | PI | *E.coli* | Urology | Stool | 14yr/Female | TEM |
| 52 | PI-Ec 25 | PI | *E.coli* | MW | Blood | 16yr/Male | TEM |
| 53 | PI-Ec 27 | PI | *E.coli* | Urology | Urine | 33yr/Female | SHV |
| 54 | PI-Ec 30 | PI | *E.coli* | Urology | Urine | 26yr/Female | SHV |
| 55 | PI-Ec 32 | PI | *E.coli* | CHW | Urine | 1yr/Female | SHV |
| 56 | PI-Ec 33 | PI | *E.coli* | ICU | Tracheal aspirate | 32yr/Male | SHV |
| 57 | PI-Ec 35 | PI | *E.coli* | MW | Urine | 22yr/Female | SHV |
| 58 | PI-Ec 38 | PI | *E.coli* | MSW | Pus | 5yr/Male | SHV |
| 59 | NH-Kp-3 | NH | *K. pneumoniae* | NICU | Urine | 8days/Male | SHV |
| 60 | NH-Kp 5 | NH | *K. pneumoniae* | ICU | Tracheal aspirate | 32yr/Male | CTX-M |
| 61 | NH-Kp 7 | NH | *K. pneumoniae* | ICU | Sputum | 1yr/Male | TEM, CTX-M |
| 62 | NH-Kp 10 | NH | *K. pneumoniae* | OPD | Urine | 58yr/Female | CTX-M, TEM, SHV |
| 63 | NH-Kp 40 | NH | *K. pneumoniae* | CHW | Urine | 1 month/Male | TEM |
| 64 | PI-Kp 9 | PI | *K. pneumoniae* | MSW | Sputum | 76yr/Male | CTX-M |
| 65 | PI-Kp 13 | PI | *K. pneumoniae* | OPD | Urine | 67 yr/ Male | CTX-M |
| 66 | PI-Kp 22 | PI | *K. pneumoniae* | SW | Urine | 44 yr/Male | CTX-M |
| 67 | NH-Pm 20 | NH | *P. mirabilis* | FSW | Urine | 2 yr/Female | CTX-M, TEM |
| 68 | NH-Cs 13 | NH | *Citrobacter* spp. | Urology | Urine | 30 yr /Female | SHV |
| 69 | NH-Cs 19 | NH | *Citrobacter* spp. | MW | Urine | 26 yr/Female | SHV |
| 70 | CH-Cs 4 | CH | *Citrobacter* spp. | NICU | Blood | 1day/Female | TEM |
| 71 | NH-Pa 13 | NH | *P. aeruginosa* | NICU | Blood | 1 month/Male | CTX-M |
| 72 | NH-Pa 17 | NH | *P. aeruginosa* | FSW | Blood | 23 yr/Female | CTX-M |
| 73 | NH-Pa 33 | NH | *P. aeruginosa* | ICU | Pus | 16yr/ Female | CTX-M |
| 74 | PI-Pa 4 | PI | *P. aeruginosa* | MSW | Wound swab | 79 yr/Male | CTX-M |
| 75 | PI-Pa 15 | PI | *P. aeruginosa* | OPD | Wound swab | 45 yr/Male | CTX-M |
| 76 | PI-Pa 23 | PI | *P. aeruginosa* | CHW | Pus | 12 yr/Female | CTX-M |

NH- Nazareth Hospital; PI – Pasteur Institute; CH- The Children Hospital; ODP- Out Patient Department; ICU- Intensive Care Unit; NICU- Neonatal Intensive Care Unit; CHW-Children Ward; MSW- Male Surgical ward; FSW- Female Surgical Ward; MW- Maternity Ward ; SW- Surgery Ward; CSF- Cerebro spinal Fluid
